# Enhancing Situational Awareness of Helicopter Pilots in Unmanned Aerial Vehicle-Congested Environments Using an Airborne Visual Artificial Intelligence Approach

**DOI:** 10.3390/s24237762

**Published:** 2024-12-04

**Authors:** John Mugabe, Mariusz Wisniewski, Adolfo Perrusquía, Weisi Guo

**Affiliations:** Faculty of Engineering and Applied Sciences, Cranfield University, College Road, Bedford MK43 0AL, UK; john.mugabe.352@cranfield.ac.uk (J.M.); m.wisnieswki@cranfield.ac.uk (M.W.); weisi.guo@cranfield.ac.uk (W.G.)

**Keywords:** situational awareness, deep learning, computer vision, stereo vision, StereoNet, Long Short-Term Memory (LSTM), threshold-based alert system

## Abstract

The use of drones or Unmanned Aerial Vehicles (UAVs) and other flying vehicles has increased exponentially in the last decade. These devices pose a serious threat to helicopter pilots who constantly seek to maintain situational awareness while flying to avoid objects that might lead to a collision. In this paper, an Airborne Visual Artificial Intelligence System is proposed that seeks to improve helicopter pilots’ situational awareness (SA) under UAV-congested environments. Specifically, the system is capable of detecting UAVs, estimating their distance, predicting the probability of collision, and sending an alert to the pilot accordingly. To this end, we aim to combine the strengths of both spatial and temporal deep learning models and classic computer stereo vision to (1) estimate the depth of UAVs, (2) predict potential collisions with other UAVs in the sky, and (3) provide alerts for the pilot with regards to the drone that is likely to collide. The feasibility of integrating artificial intelligence into a comprehensive SA system is herein illustrated and can potentially contribute to the future of autonomous aircraft applications.

## 1. Introduction

Mid-air collisions have become a frequent occurrence in the aviation industry [1]. This is observed from different recorded incidents involving UAVs and helicopters [2]. The skies are becoming increasingly congested with small airplanes, helicopters, and UAVs, which makes it challenging for pilots to track all the surrounding traffic. As a result, this increases the probability of mid-air collisions and calls for the urgent development of intelligent aerial situational awareness (SA) systems to assist helicopter pilots [3]. The existing methods rely heavily on the pilot’s vigilance and the existing traffic monitoring systems, which are not always sufficient in avoiding hazards [4]. This is because humans have limitations in how far they can see and how long their attention spans are, whilst other traditional monitoring systems have functional limitations regarding what they can do [5].

Unlike humans, AI-enabled monitoring systems are able to continuously monitor the skies by providing further situational context [6] and improving the pilot’s ability to detect and identify other nearby flying objects [7]. AI systems do not get tired and can process extremely vast amounts of visual data in real time, which makes them ideal for this application [8]. We provide the following statistics to highlight the relevance of the problem:

- Collision Incidents: According to the Federal Aviation Administration (FAA), reports of UAVs interfering with manned aircraft systems have increased. In 2020, there were 1660 drone sightings reported to the FAA by pilots, citizens, and law enforcement, with numerous near-miss incidents that involved helicopters and small planes [9].
- Safety Concerns: The European Union Aviation Safety Agency (EASA) in its 2021 annual safety review [10] reported that there is a growing concern for potential collisions between unmanned aircraft systems and other aircraft in the area as a result of increasing accessibility to these systems.
- Technological Advancements: The proliferation of new and cheaper drone technologies can cause an overwhelmed sky, potentially violating safety regulations and increasing the cognitive load on pilots [11].

In view of the above, novel integral approaches are required for increasing the situational awareness of helicopter pilots, thereby reducing the likelihood of collision and risks to people and national assets. To this end, this paper attempts to contribute to the field of aviation safety by using artificial intelligence (AI) algorithms capable of detecting and recognizing several flying vehicles which include UAVs, small planes, and helicopters. The proposed system seeks to establish the direction of travel of flying objects from different images, predict the probable collision paths, and as a result, provide timely warnings to pilots. At the core of this system is the fusion of algorithms such as YOLO for Object Detection, deep learning-based stereo vision to achieve depth estimation, Long Short-Term Memory (LSTM) for collision prediction, and a threshold-based alert system to provide a warning to the pilot for potential collision threats. The combined power of these AI systems increases the pilot’s situational awareness and reduces its cognitive load by focusing only on the detection of airborne systems.

The outline of the paper is as follows. Section 2 outlines the related work covering diverse tools for SA. Section 3 provides the methodology used in this paper, which covers the environment creation and the data collected for the training of each learning module. Section 4 describes the results obtained from the application of the proposed system. Conclusions are given in Section 5.

## 2. Related Work

Most works aimed at increasing situational awareness have focused on ground vehicles, which use an amalgamation of diverse sensing technologies including cameras, radar, and LiDAR for object detection, depth estimation, and collision avoidance [12]. The above-mentioned systems use deep learning models to detect vehicles, obstacles, and pedestrians in addition to using advanced filtering methods such as the Extended Kalman Filter (EKF) to track and predict object trajectories [13].

Using the above technologies in aviation poses significant challenges because the sky is an open environment with varying object sizes and high speeds of flying objects. This therefore requires a different treatment to the ones observed in ground vehicles. While autonomous driving systems can take advantage of multiple sensors, this paper focuses on imaging-based solutions, which are more streamlined and adaptable to existing small aircraft systems. Subsequently, we will describe the main approaches used in the literature to increase situational awareness in aviation tasks.

### 2.1. Object Detection

The first approaches for object detection relied heavily on feature-based methods such as Histogram of Oriented Gradients (HOGs) and Scale-Invariant Feature Transform (SIFT) [14]. These techniques fundamentally extracted features from images and then used machine learning classifiers such as Support Vector Machines (SVMs) to detect objects. Despite the effectiveness of these methods, they were computationally intensive and could not generalize across varied object classes and conditions [15].

Object detection is simply a computer vision technique that is used to identify and locate objects in images such as UAVs in this project. Its methods, which are based on deep learning, have transformed this field by making use of convolutional neural networks (CNNs) known for their image feature extraction capabilities to identify and classify objects with high accuracy. Two-stage detectors like R-CNN, Fast R-CNN, and Mask R-CNN first find Regions of Interest, also called RoI, in an image, and subsequently classify those regions and regress bounding boxes [15,16]. On the other hand, one-stage detectors like YOLO (You Only Look Once) bypass the RoI generation step, which allows for faster processing at the cost of some accuracy percentage [17]. Some of the recent advancements have brought onboard CenterNet, which treats objects as points and offers an excellent balance between speed and accuracy [18]. Other approaches that have been used for fire segmentation involve the use of the DeepLabv3 architecture [19], which has shown good detection results by combining it with novel vision transformer architectures.

### 2.2. Depth Estimation

Traditionally, depth estimation models have involved triangulation and stereo vision, which leverage geometry principles. Precise camera calibration and a controlled environment are two critical requirements of traditional methods that often lead to inaccuracies in complex (or dynamic) scenes. Computer stereo vision systems have been used considerably, imitating human vision to approximate the depth of objects by calculating the disparity between two images taken from slightly different viewpoints or angles [20].

**Figure 1:**
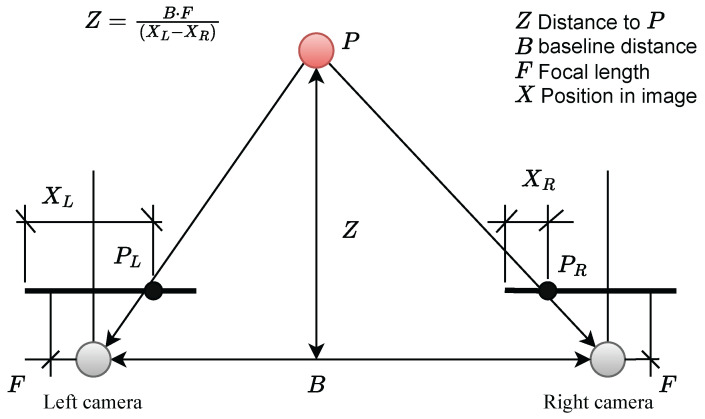
Stereo triangulation adapted from [21].

Humans use a binocular vision system which stereo vision depth estimation mimics by using two cameras to capture different viewpoints of the scene. The disparity or difference between these two images’ points allows for the calculation of depth or distance. These traditional methods, triangle similarity and computer stereo vision, have been used traditionally for this purpose [20]. Figure 1 depicts the concept of triangulation in classical computer stereo vision, in which two cameras and their corresponding image planes are placed at distance *B* from each other, and then an object *P* is captured in the images at offsets $X_{L}$ and $X_{R}$. The distance to object *P* represented by *Z* is then given by

(1) $$Z=\frac{B\cdot F}{X_{L}-X_{R}}.$$

In helicopters, specifically, the use of sensor fusion techniques for depth estimation and obstacle avoidance are utilized, as these systems utilize a combination of LiDAR, radar, and visual sensors in order to improve the accuracy of depth estimation in dynamic environments [22]. The integration of LiDAR and radar as well has been crucial for helicopters to provide reliable depth information in different weather conditions, which contributes to their safety and operational capabilities [22]. Lastly, more sophisticated depth estimation systems have been developed for real-time processing in helicopters in search and rescue missions.

StereoNet has been specifically designed to provide efficient stereo depth estimation by combining the ability of convolutional neural networks to extract features from images and the capacity of stereo vision to generate depth maps which contain object distance information. It utilizes a combination of 2D and 3D convolutions to compute disparity maps, which makes it suitable for real-time applications [23]. In [24], the StereoNet architecture combines convolutional neural networks for feature extraction and stereo vision techniques for depth estimation. It is based on the idea that a pair of stereo images taken from two different cameras are used to create an end-to-end disparity pipeline (or difference in feature position) [24], in a process that involves extracting image features using a Siamese network. StereoNet is capable of estimating the distance in areas with various nearby objects containing detailed features and textures; however, it can struggle in aerial applications due to the lack of distinct features and texture in a background that is primarily the sky.

### 2.3. Collision Prediction

Probabilistic models and filtering techniques have been traditionally relied upon to predict whether two objects will come into contact by collision. The Kalman filter and their variants have been used to track positions and predict their future states by looking at their velocities and directions [25].

LSTMs have been previously and effectively used in different UAV collision prediction cases [26]. In [27], the use of the LSTM model to predict UAV trajectories in chaotic or cluttered environments was demonstrated, which showcases their ability to handle real-time data with small computational overhead. Similarly, in [28], LSTM models were used for UAV swarm coordination, which emphasizes a success in ensuring collision-free flying.

Recent improvements, however, have further improved LSTM techniques to refine their predictive capabilities, and in order to improve accuracy and robustness, hybrid models which integrate LSTM with other machine learning techniques have been used. Those hybrid models take advantage of the benefits of LSTM and advanced ML techniques in order to yield better and more accurate predictions. One example is combining LSTM with CNN, which has been effective in applications such as traffic flow prediction and urban water demand forecasting, and has showed improved predictive capabilities [29,30].

Novel approaches have adopted the use of reservoir computing [31] for both classification and prediction of time series data. Here, the key idea is to use a reservoir with sparse connections that is left untrained to create diverse and heterogeneous high-dimensional features for better prediction results. However, the sparse connections of the reservoir can pose some stability challenges that, in the worst case, lead to the divergence of the predictions.

### 2.4. Alert System

Collision avoidance has relied on basic radar systems [32] and proximity sensors, which would trigger or initiate alerts when an object is within a threshold radius or exceeds a specific velocity, but this has led to a high number of false positives due to their inability to differentiate various obstacle types or predict future obstacle movements with accuracy. Threshold-based alert systems [33] ensure that a pilot receives the needed timely alerts without being overwhelmed with false positives. By establishing and setting appropriate thresholds, the system can balance sensitivity and specificity, which in turn provides reliable warnings while reducing unnecessary alerts. This method has been applied in Time-To-Collision (TTC) applications, where a threshold value of 10 s and a distance threshold of 100 m is used to trigger alerts, making sure that the pilot has sufficient time to take evasive action.

### 2.5. Contributions

In view of the above, we can observe that there exists a pool of AI-based tools to support pilots in navigating safely through both open and urban environments. The literature review shows that these techniques are usually applied independently such that there is an absence of a seamless integrated framework. This poses a real limitation when migrating from the simulation setup to the real-world implementation. The proposed approach aims to solve this issue by designing a complementary framework that combines the merits of object detection, depth estimation, collision prediction, and alert systems to increase the situational awareness of pilots and reduce their cognitive load. In contrast to previous approaches, the proposed framework provides a step-by-step embodied methodology that adopts a realistic simulation environment whose solution is expected to match with real-world implementations. Here, the final solution can be implemented in current UAV technologies effectively without modifying the inner subsystems. In addition, the proposed approach is scalable to novel AI algorithms that increase the accuracy of their predictions.

## 3. Methodology

**Figure 2:**
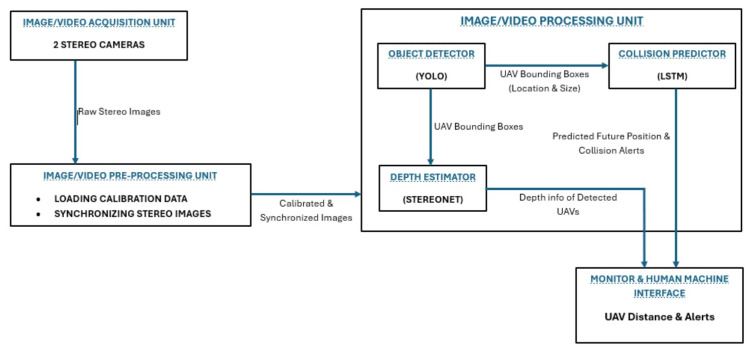
High-level architecture of the proposed airborne visual AI for SA.

The high-level architecture of the proposed system is composed by four main elements, as depicted in Figure 2: (1) Image/Video Acquisition Unit, (2) Image/Video Pre-processing Unit, (3) Image/Video Processing Unit, and (4) Monitor and Human–Machine Interface. All of these elements are described as follows.

1. Image/Video Acquisition Unit: The image and video acquisition unit is made of stereo cameras which are mounted on the helicopter, and these cameras are the ones that capture real-time video feeds of the surrounding airspace or environment. This subsystem collects image/video data and sends them to the data pre-processing unit. It consists of two stereo cameras, and they allow the execution of deep learning-based computer stereo vision, which is essential for depth estimation using the StereoNet model.
2. Image/Video Pre-processing Unit: This unit takes the captured raw image/video data acquired by the stereo cameras and carries out its initial processing. This includes using pre-calibrated data to correct distortions and carrying out synchronization to make sure that stereo images are synchronized for accurate depth estimation.
3. Image/Video Processing unit: The image and video processing unit is the core part of the entire system. This is because it contains the following modules, which are the basis of the entire system:
  - Object detection module: this module uses the YOLO algorithm to detect UAV objects in the images.
  - Depth Estimation module: this utilizes StereoNet to compute disparity maps, which are later converted to depth maps.
  - Collision prediction module: this module implements Long Short-Term Memory (LSTM) to predict likely UAV collisions based on their respective trajectories.
  - Threshold-based Alert Generation Module: this module initiates the alerts subsequent to the collision prediction made by the collision prediction module and when a distance threshold is reached.
4. Monitor and Human–Machine Interface (HMI): this serves as a visual display for detected UAVs, their distances, and collision alerts. The HMI consists of the following:
  - A visual display: this shows real-time video feeds indicating detected UAVs and their respective distances.
  - Alert indicators: these are visual and audible indicators for collision alerts.
  - Pilot interface: this allows pilots to interact with the overall system and also allows them to make any adjustments if required.

### 3.1. Helicopter Flying Environment Generation

In order to simulate an artificial intelligence system that detects UAVs, estimates their distances, and predicts the likelihood of collision, it was crucial to create an environment that adds an element of realism. Data generation was important for the training, validation, and testing of the models involved in this project. In order to generate realistic data, the environment for simulation was created by using PyBullet, Sketchfab, and Blender.

**Figure 3:**
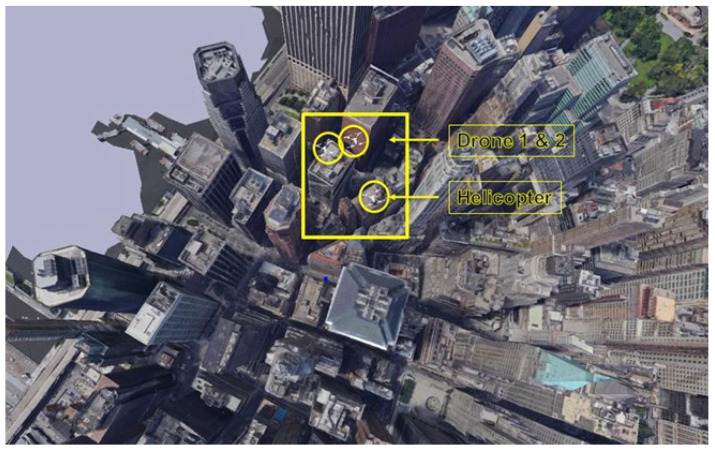
Environment design using PyBullet, Blender, and Sketchfab.

The AR.Drone was used to provide a more realistic and detailed UAV model [34]. The AR.Drone model provides a more sophisticated and accurate representation of UAVs, which includes detailed physical and visual properties that lead to realistic simulation. The AR.Drone facilitated the advanced testing of AI algorithms since it can be easily integrated into the YOLO architecture for UAV object detection. The level of detail helps to create realistic simulations necessary for the accurate detection and prediction of behaviors found in different flight scenarios. Sketchfab and Blender were used to provide a comprehensive framework for generating a high-quality 3D environment suitable for the intended goals of this paper. Figure 3 shows a simple example of the proposed simulated environment.

### 3.2. Training and Testing Data

AI algorithms require training data for the object detection, depth estimation, and path prediction elements of the proposed approach. In this paper, depth estimation and path prediction models were trained with StereoNet and LSTM models, respectively. With regards to object detection, YOLOv3-Tiny was used as the object detection model.

#### 3.2.1. StereoNet Training Data

**Figure 4:**
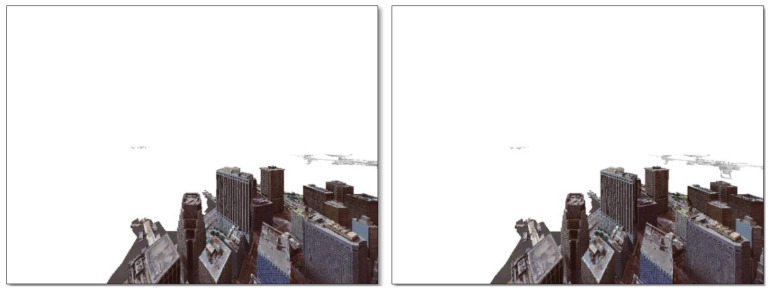
Left and right stereo image pairs.

We generated 270 images from the PyBullet environment to train the StereoNet model. The PyBullet environment was set to simulate realistic flight scenarios in which three UAVs (including one that represents the helicopter) are flying above an urban airspace. To achieve this, high-fidelity 3D models of drones and the environment landscape were imported. Then, to capture stereo images, a pair of synchronized virtual stereo cameras was set up and mounted on the simulated helicopter. We collected 90 high-resolution stereo images from the right camera and 90 high-resolution stereo images from the left camera at a rate of 30 FPS, which ensured that they were representative of the conditions needed for the depth estimation algorithm. In order to train a model that is robust and capable of generalizing in different scenarios, we created diverse simulation settings for the movement of the drones, which included circular, zigzag, and random motions across the available airspace. Figure 4 shows the left and right stereo images obtained from a simulation setting.

**Figure 5:**
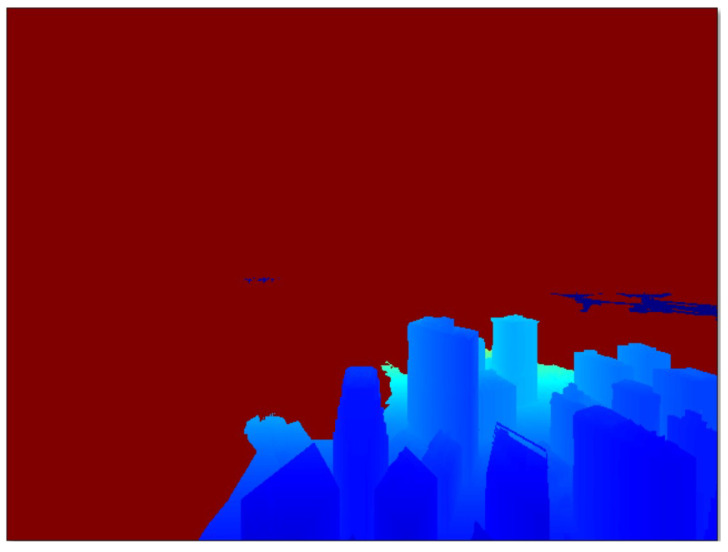
Depth map corresponding to the stereo image pairs of Figure 4.

The corresponding depth map of Figure 4 is shown in Figure 5.

The StereoNet model architecture used in this paper consists of two main components: a feature extractor and a refiner, which are key building blocks of the StereoNet model. The feature extractor uses convolutional layers to extract essential features from the stereo images taken with the camera, while the refiner combines those features in order to give the corresponding accurate depth map.

#### 3.2.2. StereoNet Testing Data

Testing and evaluation were performed using a validation set of images generated using the same PyBullet environment. This validation dataset consisted of stereo image pairs with their corresponding ground truth images.

#### 3.2.3. LSTM Training Data

The second critical part of this paper is predicting the UAVs’ future positions or paths in order to determine the probability of collision. Since the LSTM algorithm was used to perform this task for path prediction, it was trained on the UAV Delivery dataset [35]. This dataset contains the latitude, longitude, and altitude details of diverse UAV trajectories. Given the size of this dataset, which is relatively large, the latitude, longitude, and altitude columns were extracted from the log files so that they represented the *x*, *y*, and *z* coordinates of the UAVs. During data preparation, these data were normalized to improve training efficiency. The trained LSTM model could be used specifically to predict future UAVs positions, which is important for collision prediction and avoidance in this AI-based situational awareness system.

#### 3.2.4. LSTM Testing Data

A test dataset was created by splitting the original delivery dataset into training and testing subsets. The latter comprised unseen sequences that were not involved in the training process, which allowed us to prevent bias in the model evaluation.

## 4. Results

The experiments of this paper were carried out using a Dell Vostro 15 3530 Laptop machine made in China which possesses 1.70 GHz of processing power with a 13th Gen Intel (R) Core(TM) i7-1355U CPU and a 16 GB RAM.

### 4.1. Object Detection Results

This paper utilized YOLOv3-Tiny to ensure the required real-time performance in order to maintain sufficient accuracy for detecting UAVs. YOLOv3-Tiny is the lightweight network of YOLOv3, whose overall network structure is divided into three parts, namely, the feature extraction network, feature fusion network, and detection network [36]. The backbone network has 15 layers, which are only composed of convolutional layers and pooling layers, and it greatly reduces the computing overhead while also improving the detection speed [36]. The simplicity and efficiency make this algorithm suitable for the goal of this paper, which was to detect UAVs, among others goals.

**Figure 6:**
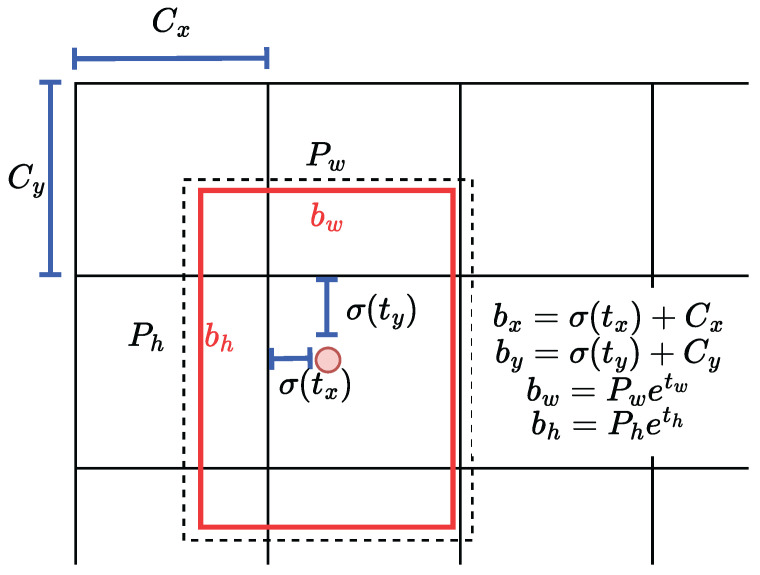
YOLOv3-Tiny bounding box regression adapted from [37].

The YOLOv3 is pre-trained on the COCO dataset, which contains a wide range of objects that include UAVs. This allowed for object detection with regards to detecting UAVs. YOLOv3 bounding box regression [37] is a result of the network calculation output, which includes the bounding box center $\left(b_{x},b_{y}\right)$, bounding box width $b_{w}$, bounding height $b_{h}$, and confidence probability of class Pr(C) [38]. The outputs of the YOLOv3 network $\left(t_{x},t_{y},t_{w},t_{h}\right)$ are transformed into the aforementioned bounding box values, where $C_{x}$ and $C_{y}$ denote the top-left coordinates, and $P_{w}$ and $P_{h}$ represent the grids’ anchors. Figure 6 shows the YOLOv3-Tiny bounding box regression transformation.

**Figure 7:**
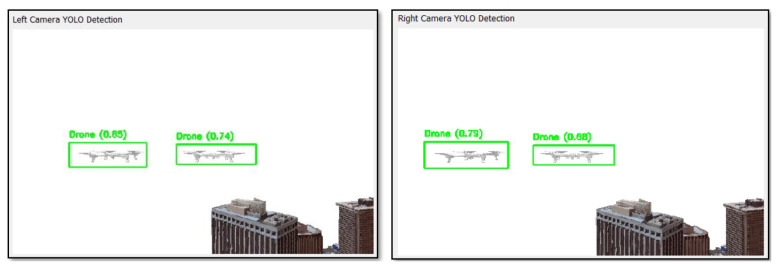
Left and right YOLOv3-Tiny UAV object detection.

YOLOv3-Tiny is integrated within the PyBullet environment, and then it takes frames from the simulation and processes them. The output is the bounding boxes drawn around the detected drones (UAVs). It should be noted that these bounding boxes are used to predict collision paths and facilitate the rest of the modules. This specific architecture achieves a mean average precision (mAP) of 33.1% on the COCO dataset for general object detection tasks. For this paper, since there is a pair of stereo cameras, object detection is performed in each camera (left and right cameras) and the bounding boxes are drawn on the detected drone. Figure 7 shows an example of the detection produced by the pre-trained YOLOv3-Tiny network in each camera.

### 4.2. Depth Estimation Results

**Figure 8:**
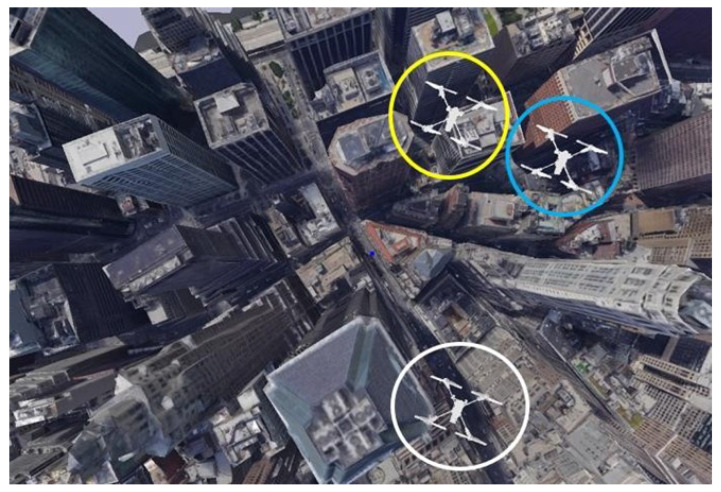
Airborne visual AI for pilots’ situational awareness PyBullet setup.

Two UAVs (Drone 1 and Drone 2) were set up to fly on a linear path towards a UAV, which simulates the helicopter (Drone 3) as depicted in Figure 8. StereoNet is used to estimate the UAV depth using the PyBullet simulation environment.

It is worth noting that the traditional methods used to solve the disparity problem rely heavily on hand-crafted features to carry out matching cost computation, cost aggregation, disparity computation, and disparity refinement. Deep learning methods such as StereoNet in this paper follow a similar procedure by leveraging CNNs to achieve more accurate disparity estimation [39]. During the implementation, the StereoNet neural network was trained on a PyBullet-generated stereo images dataset. As described, this dataset includes left and right images pairs and their corresponding depth map. We use as metrics the structural similarity index metric (SSIM) and peak signal-to-noise ratio (PSNR) to assess the quality of images in image processing tasks [40]; that is, given a reference image *f* and a test image *g*, both with size M×N, the PSNR between both images gets higher if the MSE approaches zero; conversely, a higher value of MSE reduces the PSNR, which in turn indicates differences in *f* and *g* images or lower image similarity. The PSNR is given by

(2) $$\begin{array}{cc} PSNR(f,g)= & 10\log_{10}\left(\frac{255^{2}}{MSE(f,g)}\right)\;\\ MSE(f,g)= & \frac{1}{MN}\sum_{i=1}^{M}\sum_{j=1}^{N}{\left(f_{ij}-g_{ij}\right)}^{2}. \end{array}$$

On the other hand, SSIM correlates with the quality perception of the human visual system (HVS), and it is computed by modeling image distortion as a combination of loss of correlation c(f,g), luminance distortion l(f,g)), and contrast distortion c(f,g). The SSIM is calculated as follows

(3) SSIM(f,g)=l(f,g)·c(f,g)·s(f,g).

**Figure 9:**
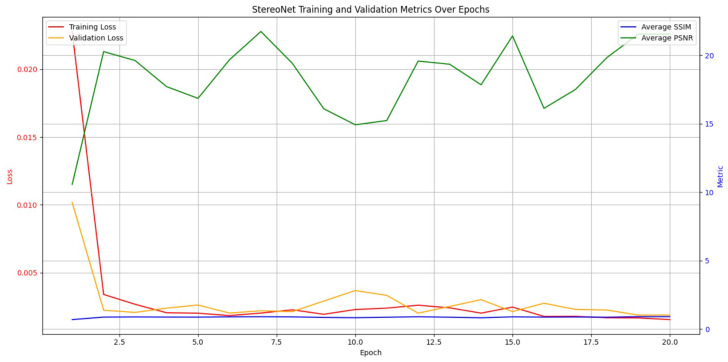
StereoNet model training evaluattion.

The training process is observed in Figure 9. In the StereoNet model training context, the stability of SSIM and PSNR suggest that the model being trained is consistently producing good-quality depth maps as it maintains the structural integrity of the image.

The inference of the trained StereoNet model is evaluated to assess the accuracy of the estimated distances. This is achieved by comparing the inference-produced depth map to the PyBullet-generated ground truth. We use the mean absolute error (MAE) and root mean squared error (RMSE) as metrics to evaluate the inference capabilities of the depth estimation model. Both the MAE and RMSE are given by

(4) $$\begin{array}{cc} \mathrm{MAE} & =\frac{1}{N}\sum_{n=1}^{n}\left|y_{i}-{\hat{y}}_{i}\right| \end{array}$$

(5) $$\begin{array}{cc} \mathrm{RMSE} & =\sqrt{\frac{1}{N}\sum_{n=1}^{n}{\left(y_{i}-{\hat{y}}_{i}\right)}^{2}}, \end{array}$$

where $y_{i}$ is the PyBullet ground truth distance value and ${\hat{y}}_{i}$ are the estimated distances using StereoNet. The obtained results are MAE = 2.8514 and RMSE = 2.8515, which reflect the precision of the model by having an error of less than 3 cm.

### 4.3. Collision Prediction and Threshold-Based Alerts

The LSTM model was trained on the UAV Delivery dataset, which was specifically designed for research in the field of Unmanned Aerial Vehicle (UAV) delivery systems. This contains different forms of data-like images, video sequences, and other sensor data which are used to develop and test algorithms for UAVs [35]. Since this dataset contains multiple attributes, the following were chosen as representative of the UAV’s *x*, *y*, and *z* coordinates: latitude, longitude, and altitude. They were fed as inputs to the LSTM model for training and the output was the predicted latitude, longitude, and altitude coordinates or future UAV positions [41]. Upon model inference, it takes as input the *x*, *y*, and *z* coordinates of UAVs in the PyBullet environment and predicts future *x*, *y*, and *z* coordinates.

**Figure 10:**
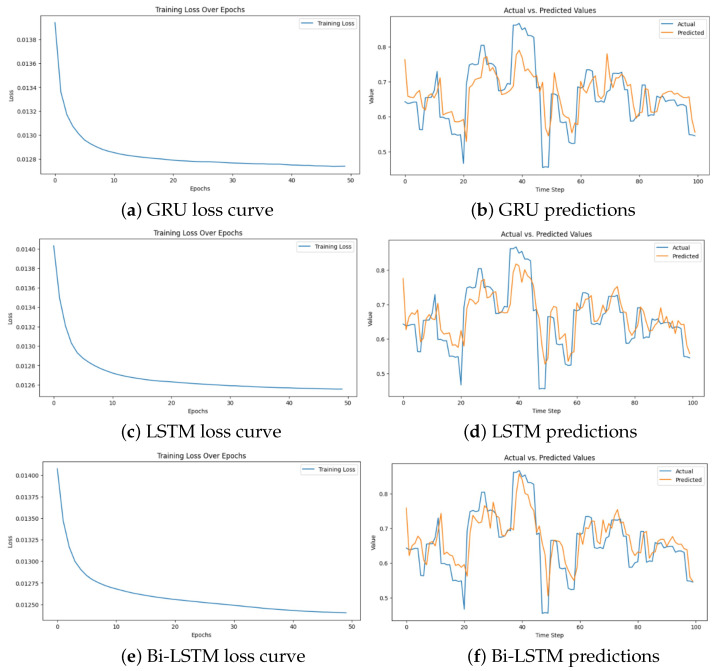
Performance of the proposed models for collision prediction.

Two more algorithms for collision prediction have been explored in order to see how they would compare with LSTM, and those models are Bi-directional LSTM and the Gated Recurrent Unit (GRU). Bi-LSTM enhances the performance of standard LSTM because it processes data in both forward and backward directions. This in effect allows it to capture context from the past and future sequences, and it is useful in predicting complex sequences, including the paths of flying objects [42]. On the other hand, GRU is a simplified version of the LSTM, whose specificity is that it combines the input and forget gates into a single update gate and makes it computationally efficient while maintaining performance in sequence prediction tasks. The comparison results are summarized in Table 1 and graphically compared in Figure 10.

The training results below suggest that the Bi-LSTM model outperforms the rest; however, it was not used because even though it has relatively better results, it is more computationally intensive while the GRU, though computationally lighter, does not achieve better accuracy results than the LSTM and Bi-LSTM models. Therefore, the optimal solution or choice for this project’s system is the Long Short-Term Memory algorithm due to its balance between accuracy and computational requirements compared to the two other algorithms.

**Figure 11:**
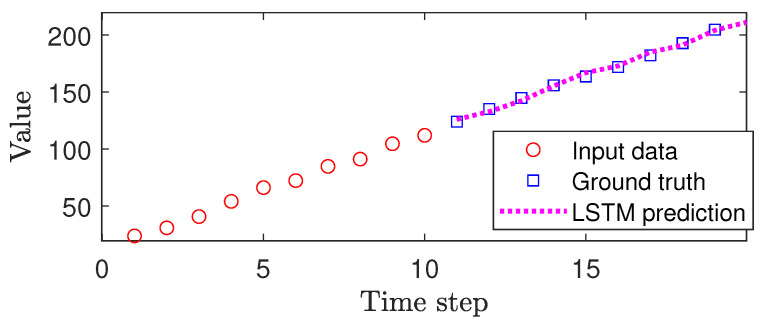
LSTM-predicted path of Drone 1 using 10 data points.

After deploying the trained LSTM model for path prediction, it takes the *x*, *y*, and *z* coordinates for the two UAVs (Drone 1 and Drone 2) and estimates their future positions against that of Drone 3, which simulates the helicopter. For each set of UAV position data, the LSTM model takes 10 data points in order to predict its future position. The position coordinates of the two UAVs are estimated from the helicopter position and the disparity value is obtained from StereoNet [43]. The model analyzes the trajectory data for all three UAVs that move in a linear path and determines whether a collision is likely to occur. Figure 11 shows a sample trajectory of Drone 1 and the predicted path given by the LSTM. Notice that the LSTM can predict accurately the future path due to the linear nature of the mission.

After this calculation, an alert threshold for the pilot is initiated, indicating that a drone is detected, along with its direction of approach and the estimated time to collision. We propose using a threshold of 3000 cm to trigger an alert so that the system is capable of performing the necessary maneuvers to avoid the collision. This threshold is heuristically selected to allow the pilot to have enough time to follow a different path. We generate the following alerts:

- *Drone Detected—Front Left! Collision in T seconds*.
- *Drone Detected—Front Right! Collision in T seconds*.

**Figure 12:**
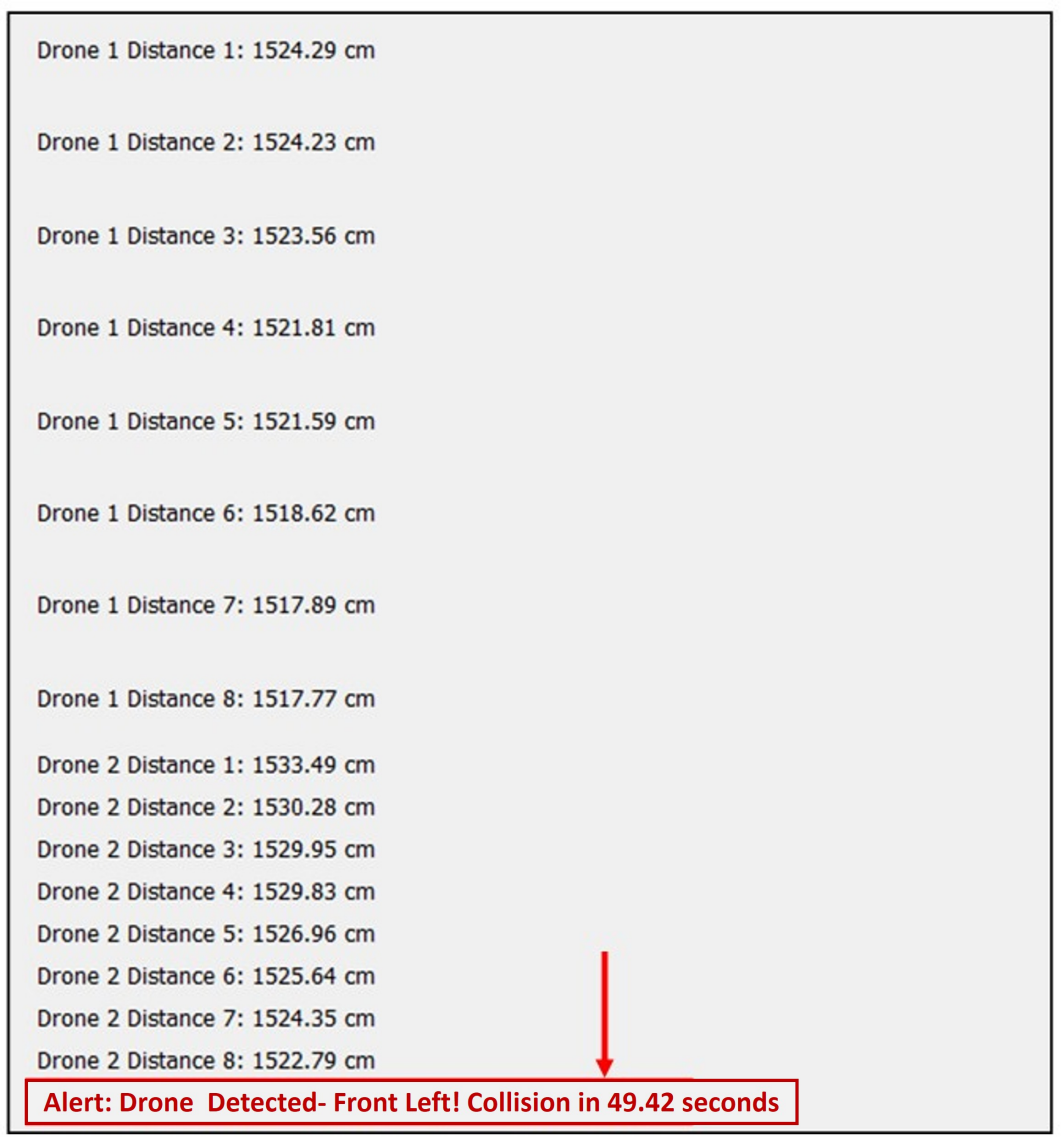
Collision alert warning to the pilot.

The above approach brings together collision prediction using the LSTM model and a threshold-based alert system based on a distance limit. In order to calculate the remaining time until collision takes place after a certain UAV was deemed likely to cross paths with the helicopter, the system uses a simple linear speed formula to estimate the time that it will take until collision takes place, i.e.,

(6) $$t=\frac{X(t)}{V(t)},$$

where *t*, V(t), and X(t) denote the estimated time to collision, the speed of the drone, and the distance between each UAV. An example of the collision alert is shown in Figure 12. Here, the alert illustrates a warning where a drone is detected from the front left side of the helicopter and the collision will take place in 49.92 s if the pilot does not take action.

**Figure 13:**
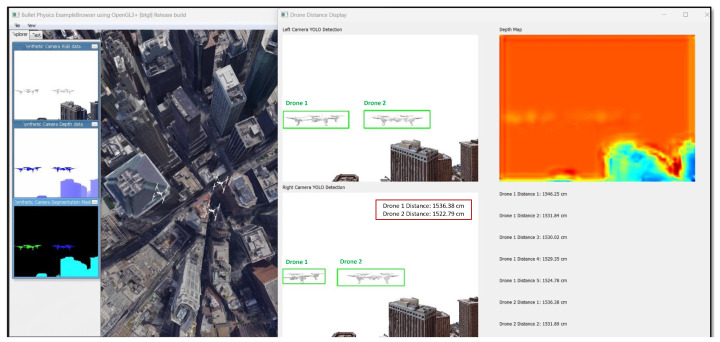
Visual representation of the overall framework.

The final framework is observed in Figure 13, which includes object detection using YOLOv3 Mini, depth estimation using StereoNet, collision prediction using LSTMs, and the threshold-based collision alert.

### 4.4. Discussion

The results presented above show how the proposed framework can integrate diverse AI tools to detect, predict, and alert pilots of potential collisions. However, there are some key points we need to consider.

- The approach uses cameras as the main sensor for the detection of other drones. Cameras are sensitive to illumination conditions, which can result in the algorithm generating many false positives, thereby increasing the cognitive load on pilots. Here, other sensor alternatives or sensor fusion techniques are required to ensure robust and stable solutions.
- The prediction algorithm is based on linear mission profiles, such that the predicted trajectory and time to collision can be estimated accurately. However, missions with different maneuvers require different treatment with a richer dataset.

The aforementioned points define some challenges and research opportunities for future work. However, this approach provides a scalable baseline for adopting different sensor modalities and prediction algorithms for situational awareness enhancement.

## 5. Conclusions

This paper proposes an airborne visual AI system for SA. The approach is based on the integration of deep learning-based object detection, stereo vision for depth estimation, and recurrent neural networks for collision prediction. The system uses YOLOv3-Tiny for object detection and StereoNet for depth estimation with accurate and fast inference results, which are key properties required for the safety of helicopters that seek to avoid collisions with nearby UAVs. In addition, a Long Short-Term Memory (LSTM) model is implemented for collision prediction. The algorithm has been demonstrated to be an effective tool for analyzing the trajectories of detected objects and generating timely alerts for pilots.

Further work will cover the sensor fusion of different perception systems such as LiDAR and RGB cameras, whereby each sensor brings onboard additional value to complement the vision-based system and depth estimation accuracy. Exploring the integration of equipment such as radar enhances the system’s performance in varying weather conditions and environments with limited visibility. Lastly, we are interested in exploring how the proposed system can be adapted for fully autonomous aircraft, where AI would not only help pilots but also play a more active role in navigation and collision avoidance.

## Figures and Tables

**Table 1:** Comparison of models for collision prediction.

Model	MAE	RMSE
GRU	0.0828	0.1292
LSTM	0.0820	0.1284
Bi-LSTM	0.0811	0.1281

## Data Availability

No new data were created or analyzed in this study. Data sharing is not applicable to this article.

## Abbreviations

The following abbreviations are used in this manuscript:

AI	Artificial Intelligence
Bi-LSTM	Bi-Directional Long Short-Term Memory
CNN	Convolutional Neural Network
EFA	European Aviation Safety Agency
ESA	European Space Agency
FAA	Federal Aviation Administration
FPS	Frames Per Second
GDPR	General Data Protection Regulation
GPU	Graphics Processing Unit
GRU	Gated Recurrent Unit
HMI	Human Machine Interface
LSTM	Long Short-Term Memory
MAE	Mean Absolute Error
RGB	Red Green Blue
RNN	Recurrent Neural Network
SIFT	Scale-Invariant Feature Transform
SSD	Single Shot Multi-box Detector
SVM	Support Vector Machine
TTC	Time-To-Collision
URDF	Unified Robot Description Format
UAV	Unmanned Aerial Vehicle
YOLO	You Only Look Once
